# An online acceptance, commitment, and self-compassion based treatment to decrease psychological distress in people with type 2 diabetes: A feasibility randomised-controlled trial

**DOI:** 10.1016/j.invent.2023.100658

**Published:** 2023-08-08

**Authors:** Ayşenur Kılıç, Joanna Hudson, Whitney Scott, Lance M. McCracken, Ruth A. Hackett, Lyndsay D. Hughes

**Affiliations:** a School of Pharmacy, University College London, London WC1H 9JP, UK; b Department of Psychology, King's College London, London SE1 9RT, UK; c INPUT Pain Management Unit, Guy's and St Thomas' NHS Foundation Trust, London SE1 7EH, UK; d Department of Psychology, Uppsala University, Uppsala 751 42, Sweden

**Keywords:** Diabetes mellitus, Type 2, Psychological flexibility, Self-compassion, Randomised controlled trial

## Abstract

**Background and purpose:**

This study explored the feasibility and acceptability of conducting a larger trial of a self-guided, online self-compassion and acceptance and commitment therapy (ACT) focused treatment among people with type 2 diabetes (T2D) to decrease psychological distress.

**Materials and methods:**

This study was a two-arm, parallel, feasibility randomised controlled trial with nested qualitative methods. UK adults with T2D were randomly (1:1) allocated to a five-week online self-compassion and ACT treatment or waitlist control. Information regarding recruitment, trial retention, and treatment completion was collected, and post-treatment semi-structured interviews were conducted to assess feasibility and acceptability. Self-report measures of psychological distress (depression, anxiety, diabetes distress) and potential treatment processes (self-compassion and psychological flexibility) were completed as secondary feasibility outcomes.

**Results:**

Fifty-five (60.44 %) out of 91 people who accessed the study link were eligible to participate. Of these, 33 eligible participants (60 %) were randomly assigned to treatment (*n* = 19) or control arms (waitlist; *n* = 14). While treatment completion was 47.37 %, trial retention rates were 39.39 % (5-week follow-up) and 21.2 % (9-week follow-up). Secondary feasibility outcomes of treatment effect estimates are difficult to interpret in light of low treatment completion and trial retention rates.

**Conclusion:**

A larger trial of the self-guided, online self-compassion treatment to decrease psychological distress in people with T2D may be beneficial, but it has limited feasibility in its current form. Further efforts are needed to improve treatment acceptability of online self-compassion and ACT focused treatment and trial procedures.

## Highlights

• Self-compassion may decrease psychological distress in people with type 2 diabetes (T2D).
• Self-compassion and acceptance and commitment therapy (ACT) can be combined.
• ACT and self-compassion for people with T2D may decrease psychological distress.
• Study findings can improve treatment and trial procedures for future research.

## 1 Introduction

4.7 million people affected by diabetes in the UK alone (Diabetes UK, 2019a) with 90 % of those cases accounted for by type 2 diabetes (T2D) (International Diabetes Federation, 2019). Around one in three people with diabetes experience at least one form of psychological distress, such as depression, anxiety or diabetes specific distress (Mommersteeg et al., 2013; Smith et al., 2013), which negatively impact on blood glucose levels, treatment, and illness progression (Garrett and Doherty, 2014). Therefore, the treatment of comorbid psychological distress is a crucial part of T2D management (Donald et al., 2013). However, the currently available treatment methods for comorbid psychological distress in diabetes is suboptimal (Diabetes UK, 2019b).

In England, Improving Access to Psychological Therapies (IAPT) services provide face-to-face and online Cognitive Behavioural Therapy (CBT) for long-term conditions, including diabetes (Wroe et al., 2015; National Collaborating Centre for Mental Health, 2018). However, CBT may not benefit all people, especially those who are highly self-critical, where relapses in mental health problems are common (Rector et al., 2000). For example, being directed to reappraise ‘maladaptive’ cognitions through CBT can be viewed as another source of criticism directed to oneself, further fostering the vicious cycle of criticism (Yadavaia et al., 2014). Similarly, a recent meta-analysis exploring the efficacy of CBT-based interventions for people with diabetes found that even though CBT had significantly large effects on decreasing depression, small and no effects were found for anxiety and distress, respectively (Li et al., 2022). Therefore, CBT may be beneficial, particularly for depression, but may not be the best fit for all people or different outcomes (e.g., anxiety; Jenkinson et al., 2022), and new treatment approaches are needed to address this gap.

Living with diabetes may increase one's experience of self-criticism (Friis et al., 2015) as diabetes is a stigmatised condition. Nearly half of the people with diabetes feel they are blamed by others for being the cause of the condition or its progression (e.g., over-eating; Ringel and Ditto, 2019), leading to increased self-criticism. Further, people with diabetes can be exposed to social monitoring, where one's health-related behaviours are judged or criticised by others (e.g., Yang et al., 2016) if it is not in line with the recommendations resulting in an increased focus on one's self-management failures. Considering the daily challenges of maintaining diabetes, such as monitoring blood glucose, food consumption or physical activity levels, personal failures can be experienced on frequent basis, which may be particularly likely to increase one's self-directed criticism (Friis et al., 2016). Therefore, alternative approaches may be needed to optimise psychological distress management in people with T2D, especially for self-criticism.

Treatments focused on enhancing self-compassion may be particularly useful to counteract self-criticism to support psychological distress management in people with T2D. Self-compassion describes kindness, mindful awareness, and recognition of the common humanity in suffering, rather than self-criticism, over-identification with suffering, and isolation (Neff, 2003). A recent systematic review of randomised-controlled trials (RCT) indicates that self-compassion interventions may be beneficial for people with chronic physical health conditions, including one RCT in diabetes (Kılıç et al., 2021). According to the study, completion of a group-based Mindful Self-Compassion Course (MSC) (Germer and Neff, 2019) was associated with psychological and physiological wellbeing, such as decreased psychological distress and HbA1c outcomes compared to a waitlist control. However, despite these promising findings, few RCTs have investigated the role of self-compassion in diabetes.

Although self-compassion comes from a distinct theoretical model, psychological flexibility is related to self-compassion and consists of present-moment-awareness, openness to difficult experiences, and engagement with valued life activities in the presence of challenges (Hayes et al., 2011). Psychological flexibility is the key process targeted within Acceptance Commitment Therapy (ACT) (Hayes et al., 2011) and there is growing evidence that ACT is useful for people with long-term conditions (Graham et al., 2016), which may also increase self-compassion. There is also emerging evidence that psychological flexibility may benefit people with T2D (Nicholas et al., 2021), and be more applicable for treating coexisting mental health problems, where CBT may be limited (Arch and Ayers, 2013). Thus, treatments that specifically aim to increase self-compassion and psychological flexibility may improve psychological outcomes in people with T2D.

This study aimed to evaluate the feasibility of conducting a larger trial of an online treatment that included elements of MSC and ACT for psychological distress (depression, anxiety, and diabetes-specific distress) among people with T2D. The feasibility of a larger trial was assessed in terms of recruitment, treatment completion, and trial retention rates and qualitative feedback about the treatment. Secondarily, estimates of effect for self-reported psychological distress outcomes and treatment process variables were collected to ascertain proof of concept if deemed feasible.

## 2 Material and methods

This study was designed as an online, two-arm, parallel randomised-controlled feasibility trial with nested qualitative methods. Adults with T2D were randomised to receive the online Acceptance, Commitment, and Self-Compassion based treatment in Diabetes (ACSBT-D) or to the waiting list condition. The CONSORT checklist (Schulz et al., 2010) (Appendix A) and JARS guidelines (American Psychological Association, 2008) were applied in reporting the findings. The study protocol was approved by King's College London (Ethics Reference Number: HR-20/21-19630).

### 2.1 Recruitment of participants and baseline assessment

Eligibility criteria included adults (aged ≥18) in the UK with a self-confirmed clinical diagnosis of T2D. Participants had to be able to read and write English and needed access to the Internet. In addition, participants who reported a severe mental health problem (e.g., bipolar disorder) at the time of screening were also excluded from the study. Given limited research on treatments targeting self-compassion and psychological flexibility in T2D, specific inclusion/exclusion criteria were not set in terms of specific levels of distress (depression, anxiety, or diabetes-specific distress).

Study advertisements ran on social media platforms such as Facebook, Instagram and patient forums (e.g., Diabetes UK). Participants were recruited between 11th January and 31st May 2021. The last follow-up assessment was collected on 18th July 2021. All participants provided online informed consent and completed a baseline assessment, including providing demographic and medical information and self-report questionnaires before randomisation. The study used the Qualtrics Survey Platform (Qualtrics, Provo, UT) for data collection (baseline, and five-week and nine-week follow-ups). Qualtrics was also used for the delivery of the ACSBT-D.

### 2.2 Randomisation, concealment, and blinding

Study participants were randomly allocated to one of two groups by Qualtrics Randomiser using a 1:1 allocation ratio. Both participants and the research team were blind to the allocation at the time of randomisation. However, given the nature of the treatment, one researcher (AK) who provided access to the ACSBT-D programme and follow-up assessment questionnaires was unblinded from the point of allocation onwards. However, the remote completion of standardized online questionnaires by participants was used to minimise bias.

### 2.3 Patient involvement activity

Six people with T2D were involved in several stages of the trial by giving feedback on treatment material and the trial methods. Prior to the trial set up, those people attended an hour-long-semi-structured interviews to provide their input. In the first part of the interview, patient representatives viewed a weekly session of the treatment manual. They have commented on the material and treatment procedures. In the second part of the interview, patient representatives answered questions about the conduct of the trial (see Appendix B for how patient input influenced decisions about treatment and trial procedures).

### 2.4 Intervention and comparator

#### 2.4.1 ACSBT-D

ACSBT-D programme was developed by applying the Medical Research Council framework for developing complex interventions (Skivington et al., 2021). Accordingly, consecutive studies carried out for intervention development, which included a systematic review (Kılıç et al., 2021) and a longitudinal assessment of self-compassion and psychological flexibility among people with T2D (Kılıç et al., 2022). Based on the findings of these earlier studies, the primary author developed the ACSBT-D program and carried out patient involvement activities. Accordingly, the treatment programme integrated psychological flexibility and self-compassion concepts from ACT (Hayes et al., 2011) and MSC (Germer and Neff, 2019). The treatment consisted of five-weekly sessions delivered online (Qualtrics), each approximately 30 min in duration. The sessions focused on developing acceptance, commitment, and self-compassion and self-care in diabetes (Appendix C and D for learning aims and targeted mechanism of change). The material included metaphors, experiential, mindfulness, and compassion practices which were presented in visual and audio formats for improved accessibility. Participants were also advised to complete 10–15 min of optional daily practice to strengthen learning from the weekly sessions. An optional homework booklet was provided at the start of the treatment programme for this purpose. The treatment was entirely self-directed, and no therapist support was provided.

#### 2.4.2 Waitlist control

Participants allocated to the waitlist continued their current medical treatment and did not receive any information regarding the ACSBT-D programme. Following completion of five and nine-week follow-ups, they received weekly access to the treatment material. This control condition does not permit inferences about whether any possible treatment effects are due to non-specific (e.g., expectations for treatment) versus specific mechanisms (i.e., self-compassion and psychological flexibility). However, this provides a more robust design than a single-arm study in which changes observed during treatment could be related to natural fluctuations in symptoms over time or regression to the mean. A waitlist control was deemed suitable given the preliminary nature of research into this form of treatment in T2D.

### 2.5 Follow-up

Follow-up data were collected between February and July 2021. Participants were asked to complete self-report questionnaires at five- and nine-weeks post-randomisation. The five-week follow-up reflects the end of treatment assessment. The nine-week follow-up was selected to determine the feasibility of short-term follow-up assessment and whether any possible treatment effects might be maintained in the short-term. Participants received the questionnaire link by email and completed via Qualtrics.

### 2.6 Interviews

After the nine-week follow-up, participants were invited to an optional semi-structured interview (Appendix E for topic guide) lasting 15 min to discuss their views of ACSBT-D with one researcher (AK). Interviews were used to understand intervention engagement barriers, acceptability, and satisfaction with the intervention and trial methods.

### 2.7 Outcome measures

As mentioned, the primary focus of this study was to understand the feasibility of conducting a larger RCT to evaluate the efficacy of the treatment programme by collecting information regarding trial recruitment and retention, and treatment completion. A secondary objective was to estimate possible change in key self-reported treatment outcome and process variables. Key secondary outcome variables included depression, anxiety, diabetes-distress, well-being, diabetes-related quality of life, and diabetes self-management. Key secondary process variables included self-compassion and psychological inflexibility. Appendix F summarizes the questionnaires used to assess these variables. All of the included questionnaires are standardized and well-validated.

### 2.8 Sample size

As this was a feasibility trial, a formal sample size calculation was not required (Thabane et al., 2010). A target sample size within the region of 40 (20 per arm) was set with a planned baseline recruitment period of five months. This is consistent with guidance provided about the minimum participants needed per arm in feasibility studies (Whitehead et al., 2016). As a key objective was to determine recruitment feasibility, and the recruitment rate was unknown, we aimed for a minimum of 12 per group (Julious, 2005) in the case that recruitment was less than expected. To obtain qualitative data about treatment and trial acceptability, it was planned to interview at least five people from the treatment group at the end of their participation.

### 2.9 Statistical analysis and feasibility criteria

Descriptive statistics were computed to describe characteristics of the sample. The primary feasibility parameters were recruitment, treatment completion, and follow-up retention rates. The recruitment rate was computed as the number of people who accessed the study link and were eligible relative to people who completed the baseline assessment and were randomised. A previous trial for an internet-based self-management program for T2D suggested that a recruitment rate of 37 % is reasonable and exceeds that typically found in face-to-face diabetes self-management programmes (Glasgow et al., 2010); therefore, 37 % was the threshold against which the current recruitment rate was judged.

Treatment completion was computed based on the number of sessions completed, expressed as the median (interquartile range [IQR]) and frequencies and percentages). Treatment completion was defined as completing at least three ACSBT-D sessions (more than half), which was sufficient for participants to be exposed to key therapeutic content. The proportion of treatment completion in online self-management programmes for chronic disease has varied widely (between 9 and 82 %), with a pooled estimate of 43 % treatment drop-outs, indicating 57 % treatment completion across studies (Meyerowitz-Katz et al., 2020). Therefore, 57 % was selected as a threshold against which to judge the treatment completion rate. Trial retention was computed as the number of people who completed follow-up assessments (5 and 9 weeks) relative to the number that completed baseline assessment. The threshold for trial retention to be feasible was judged against 57 %, which was the pooled attrition rate of app-based interventions for chronic diseases (Meyerowitz-Katz et al., 2020). Reasons for dropouts and adverse events were recorded. The acceptability of the intervention, including possible adverse events, and trial methodology was explored qualitatively using open-ended questions. Interviews were recorded, transcribed, and analysed using inductive content analysis.

SPSS Statistics 27 (IBM Corp., 2020) was used for analyses. Independent sample *t*-tests and Chi-square tests were computed for demographics and baseline outcome and process variables to examine randomisation success. Independent sample t-tests and Chi-square tests were also performed to explore differences between treatment and follow-up completers and non-completers. For the secondary outcome variables of possible treatment effects, no significance testing was reported as it was not the purpose of this study to assess efficacy and it was not powered to do so, as mentioned. Only within group effect sizes for baseline to 5 weeks (T1 – T2), 5 weeks to 9 weeks (T2 – T3), and then baseline to 9 weeks (T1 – T3) were computed by calculating Hedge's *g* (corrected for small sample size) and 95 % confidence intervals (CIs) were reported to examine the possible direction of effects for the treatment and the uncertainty around the effects based on the width of the CIs (Eldridge et al., 2016). Effect sizes interpreted as small (0.20), medium (0.50), and large (0.80) (Cohen, 1988).

## 3 Results

### 3.1 Primary feasibility outcomes

#### 3.1.1 Recruitment and participant characteristics

**Fig. 1:**
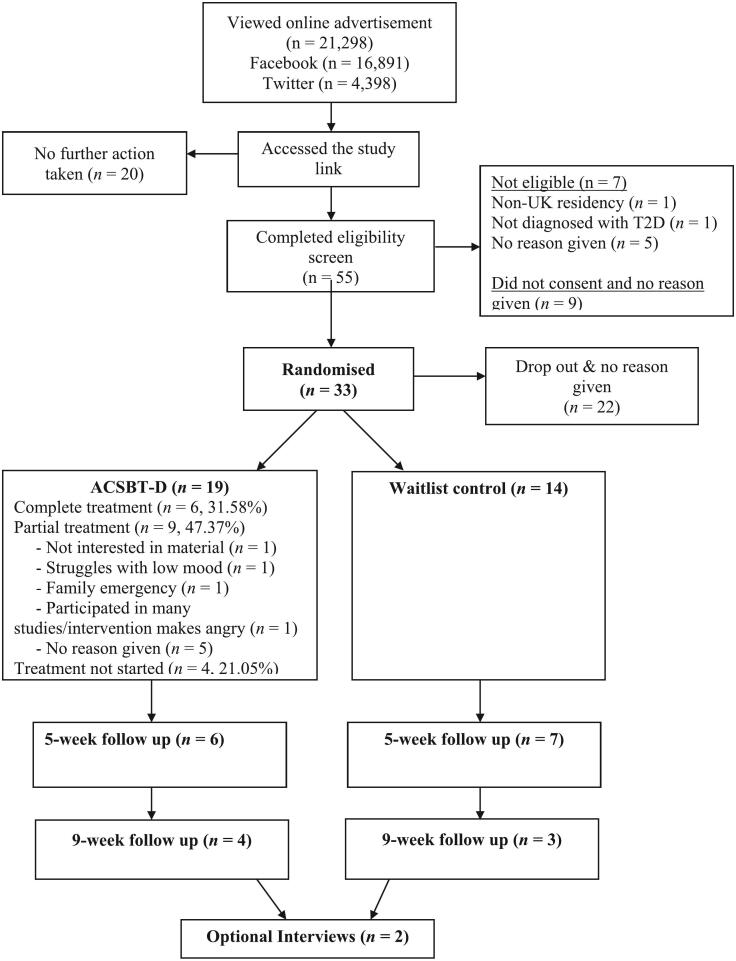
Study flow diagram.

Ninety-one people accessed the study link and, of these, 55 (60.44 %; 95 % CI [49.64 %, 70.54 %]) were eligible to participate (Fig. 1). Thirty-three eligible participants (60 %; 95 % CI [45.91 %, 72.98 %]) completed the baseline measurements and were randomised (19 to ACSBT-D, 14 to waitlist; recruitment rate 36 %). The study sample included predominantly women (84.85 %) of white ethnicity (84.9 %). The mean age of the sample was 55.85 (*SD* = 10.34) years, and most participants were married or in a civil partnership (*n* = 20; 60.6 %). The mean age of leaving full-time education was 20.81 years (*SD* = 5.80), and most participants were employed (*n* = 21; 63.5 %). The mean length of T2D diagnosis was 10.24 years (*SD* = 6.57). Further medical information about the sample is shown in Table 1.

**Table 1:** Characteristics of the study participants at baseline (N = 33).

	Treatment arm	Waitlist arm	Total sample
	Mean (SD)/n(%)	Mean (SD)/n(%)	Mean (SD)/n(%)
Sex			
Women	16 (84.2 %)	12 (85.6 %)	28 (84.8 %)
Men	3 (15.8 %)	2 (14.2 %)	5 (15.1 %)
Age	57 (10.27)	54.28 (10.60)	55.85 (10.34)
Age of leaving full-time education	20.41 (4.44)	21.29 (7.27)	20.81 (5.80)
Ethnicity			
White	17 (89.4 %)	11 (78.6 %)	28 (84.9 %)
Mixed Background	1 (7.1 %)	1 (7.1 %)	2 (3 %)
Asian/Asian British	2 (14.3 %)	1 (5.3 %)	3 (9.1 %)
Relationship status			
Single	2 (10.5 %)	2 (14.3 %)	4 (12.1 %)
Married/ civil partnership	12 (63.2 %)	8 (57.1 %)	20 (60.6 %)
Widowed	–	1 (7.1 %)	1 (3 %)
Co-habiting	3 (15.8 %)	–	3 (9.1 %)
Separated/Divorced	2 (10.5 %)	3 (21.4 %)	5 (15.2 %)
Work status			
Employed	13 (68.5 %)	8 (57.1 %)	21 (63.5 %)
Unemployed	1 (7.1 %)	1 (5.3 %)	2 (6.1 %)
Student	–	1 (7.1 %)	1 (3 %)
Retired	5 (26.3 %)	4 (28.6 %)	9 (27.3 %)
Length of T2D (in years)	12.26 (6.35)	7.5 (6)	10.24 (6.57)
Body mass index	31.93 (5.62)	33.43 (6.82)	32.58 (6.10)
Activity level			
Inactive (>30 min a week)	4 (21.1 %)	4 (28.6 %)	8 (24.2 %)
Moderately active (30–60 min a week)	7 (36.8 %)	7 (50 %)	14 (42.4 %)
Active (60–150 min a week)	8 (42.1 %)	3 (21.4 %)	11 (33.3 %)
Number of comorbid physical conditions			
0	4 (23.5 %)	3 (21.4 %)	7 (22.6 %)
1–3	11 (64.6 %)	10 (71.4 %)	21 (67.8 %)
4–7	2 (11.8 %)	1 (7.1 %)	3 (9.7 %)
Previous psychological treatment (Yes)	10 (52.6 %)	7 (50 %)	17 (51.5 %)

Note. SD: Standard deviation; n: number of people; %: percentage.

Threshold scores for depression, anxiety and diabetes distress indicated that participants were not depressed (<10) (Kroenke et al., 2009) or mildly anxious (<5) (Spitzer et al., 2006) on average. However, participants were experiencing severe diabetes distress (>40) (Welch et al., 1997). Participants who only completed the baseline questionnaires appeared similar to people who also completed follow-up questionnaires in terms of demographic characteristics, distress, self-compassion, and psychological flexibility (*p* > 0.05).

#### 3.1.2 Treatment completion and trial retention

A total of 9 of 19 participants (47.37 %; 95 % CI [24.45 %, 71.14 %]) randomised to ACSBT-D programme were considered treatment completers. The median number of completed sessions by the treatment group was 2 (IQR = 4). Six of 19 participants (31.58 %; 95 % CI [12.58 %, 56.55 %]) completed all the treatment sessions. Reasons given for non-completion were not being interested in the material (*n* = 1), being too busy to participate (*n* = 1), ongoing low mood unrelated to the treatment (*n* = 1), family emergency (*n* = 1), taking part in too many studies/treatment made participant angry (*n* = 1), and no reason given (*n* = 5).

Of the 19 participants allocated to ACSBT-D condition, 6 (31.6 %; 95 % CI [12.58 %, 56.55 %]) and 4 (21.1 %; 95 % CI [6.05 %, 45.57 %]) completed five- and nine-week follow ups, respectively. Of the 14 people allocated to the waitlist, 7 (50 %; 95 % CI [23.04 %, 76.96 %]) and 3 (21.4 %; 95 % CI [4.66 %, 50.80 %]) completed the five-week and nine-week assessments. The combined retention was 13 (39.39 %; 95 % CI [22.9 %, 57.9 %]) and 7 (21.2 %; 95 % CI [8.98 %, 38.9 %]) for assessments points at five- and nine-weeks.

### 3.2 Qualitative assessment of treatment and trial acceptability

Although ten people were contacted for the post-treatment interviews, only two consented to participate in these. No reason was provided for not participating in the interviews. The most-reported helpful aspects of the ACSBT-D according to those interviewed were that it was well structured and easy to understand, included interesting topics (e.g., values), and the self-compassionate perspective for diabetes management was considered advantageous. Being present and accountable for one's actions and having adequate time to complete each session were also considered strengths. The most-reported least helpful aspect of the ACSBT-D was the lack of face-to-face interaction/group setting, which may relate to feeling distant and a sense of not belonging. Some materials were found to be too easy for advanced practitioners of mindfulness (Appendix G).

Two adverse events were reported. One participant indicated that training material made them angry. This participant also reported feeling overburdened due to participating in multiple research projects simultaneously. Another participant reported experiencing severe low mood, resulting in limited daily activities (e.g., staying in bed) unrelated to participating in the training.

### 3.3 Secondary feasibility outcomes

Study randomisation was successful such that there were no baseline differences between groups on study variables (*p* > 0.05). Within the ACSBT-D group, medium improvements were observed from baseline to five-weeks for diabetes distress and self-compassion, and small effects for depression and anxiety symptoms, psychological inflexibility, and diabetes well-being. From baseline to nine-weeks, improvements were large for self-compassion, medium for distress, small for depression, anxiety, and well-being, and less than small for psychological inflexibility. In contrast, within the waitlist group, from baseline to five-weeks, small improvements were observed for diabetes distress, self-compassion, and psychological inflexibility, while less than small effects were seen for the other variables. From baseline to nine-weeks in the waitlist, improvements were large for well-being, medium for diabetes distress, psychological inflexibility and QoL, and small for depression, anxiety, and self-compassion. However, across both groups and time-points the confidence intervals were wide indicating that the true effects may range between small to large harms and small to large benefits (Table 2, Table 3).

**Table 2:** Descriptive statistics for secondary outcomes at baseline, 5-weeks and 9-weeks follow-ups for ASBCT-D and waiting list study arms.

		Treatment arm		Waitlist arm	
Self-report measures		Mean (SD)	Median (Interquartile range)	Mean (SD)	Median (Interquartile range)
Depression (PHQ-8)	Baseline	7.58 (4.84)	8 (0–17)	8.23 (4.26)	7 (3–18)
	5-weeks follow up	5.33 (3.61)	4.5 (1−11)	8.50 (4.49)	8 (3–15)
	9-weeks follow up	6 (6.38)	4.5 (0–15)	6.33 (5.13)	5 (2−12)
Anxiety (GAD-7)	Baseline	5 (3.90)	4 (0−12)	4.23 (2.83)	4 (0–8)
	5-weeks follow up	3.50 (2.59)	4 (0–6)	4.67 (4.72)	2.5 (0–12)
	9-weeks follow up	3 (4.76)	1 (0−10)	5 (5.20)	2 (2−11)
Diabetes distress (PAID)	Baseline	42.50 (16)	43.75 (11.25–70)	45.42 (19.86)	52.50 (15–70)
	5-weeks follow up	32.29 (20.08)	25 (13.75–67.50)	38.75 (25.71)	33.12 (12.50–82.50)
	9-weeks follow up	31.25 (19.20)	33.75 (10–47.50)	32.92 (23.13)	32.50 (10–56.25)
Wellbeing (W-BQ12)	Baseline	19.42 (6.87)	17 (6–33)	19.50 (3.72)	20 (11–25)
	5-weeks follow up	21.33 (3.61)	21.50 (17–27)	19.33 (5.39)	19.50 (12–27)
	9-weeks follow up	22.25 (4.11)	23 (17–26)	25.33 (6.66)	27 (18–31)
Diabetes QoL (ADDQoL-19)	Baseline	−1.69 (0.88)	−1.74 ([−3.37] – [−0.16])	−2.60 (2.38)	−1.94 ([−6.47] – 1.17)
	5-weeks follow up	NA	NA	NA	NA
	9-weeks follow up	−0.90 (0.72)	−1.02 ([−1.63] – 0.05)	−1.11 (1.57)	−0.7 ([−2.84] – 0.21)
Diabetes self-management (DSMQ)	Baseline	6.17 (2.11)	6.04 (1.67–8.96)	7.29 (0.88)	7.18 (6.04–8.72)
	5-weeks follow up	NA	NA	NA	NA
	9-weeks follow up	7.23 (1.71)	6.88 (5.83–9.33)	7.23 (1.73)	8.12 (5.83–9.23)
Self-compassion (SCS)	Baseline	2.69 (0.58)	2.68 (1.36–3.63)	2.75 (0.41)	2.73 (2.13–3.40)
	5-weeks follow up	2.97 (0.48)	2.92 (2.32–3.58)	2.91 (0.58)	2.86 (2.28–3.59)
	9-weeks follow up	3.28 (0.98)	3.40 (1.98–4.35)	2.97 (0.76)	2.78 (2.33–3.81)
Psychological inflexibility (AAQ-2)	Baseline	20.84 (9.46)	20 (7–36)	23.15 (6.26)	23 (13−32)
	5-weeks follow up	16.67 (5.64)	17.50 (10−22)	20.67 (11.13)	16.50 (10–37)
	9-weeks follow up	20.67 (8.50)	21 (12–29)	19 (11.53)	15 (10−32)

Note. AAQ-2: Acceptance and Action Questionnaire-2; ADDQoL19: Audit of Diabetes Dependent Quality of Life 19; GAD-7: Generalised Anxiety Disorder-7; PAID: Problem areas in Diabetes; PHQ-8: Patient Health Questionnaire-8; SCS: Self-Compassion Scale; W-BQ12: Well-Being Questionnaire.

**Table 3:** Within-groups effect sizes on secondary outcome variables.

Variables	T1 – T2	T2 – T3	T1 – T3
Depression			
Treatment arm	0.47, 95 % CI [−0.46, 1.40]	−0.13, 95 % CI [−0.46, 1.40]	0.30, 95 % CI [−0.78, 1.38]
Waitlist arm	−0.06, 95 % CI [−1.03, 0.91]	0.41, 95 % CI [−0.99, 1.80]	0.41, 95 % CI [−0.85, 1.67]
Anxiety			
Treatment arm	0.40, 95 % CI [−0.46, 1.40]	0.13, 95 % CI [−1.14, 1.39]	0.48, 95 % CI [−0.61, 1.56]
Waitlist arm	−0.12, 95 % CI [−1.09, 0.85]	−0.06, 95 % CI [−1.45, 1.33]	−0.23, 95 % CI [−1.49, 1.03]
Diabetes-distress			
Treatment arm	0.58, 95 % CI [−0.35, 1.51]	0.05, 95 % CI [−1.22, 1.31]	0.66, 95 % CI [−0.44, 1.75]
Waitlist arm	0.29, 95 % CI [−0.69, 1.28]	0.21, 95 % CI [−1.18, 1.60]	0.58, 95 % CI [−0.71, 1.86]
Self-compassion			
Treatment arm	−0.50, 95 % CI [−1.49, 0.50]	−0.36, 95 % CI [−1.69, 0.96]	−0.87, 95 % CI [−1.98, 0.24]
Waitlist arm	−0.33, 95 % CI [−1.30, 0.65]	−0.08, 95 % CI [−1.47, 1.30]	−0.44, 95 % CI [−1.70, 0.83]
Psychological inflexibility			
Treatment arm	0.46, 95 % CI [−0.47, 1.39]	−0.54, 95 % CI [−1.95, 0.87]	0.02, 95 % CI [−1.20, 1.24]
Waitlist arm	0.30, 95 % CI [−0.68, 1.27]	0.13, 95 % CI [−1.26, 1.52]	0.54, 95 % CI [−0.73, 1.81]
Wellbeing			
Treatment arm	−0.29, 95 % CI [−1.21, 0.63]	−0.22, 95 % CI [−1.49, 1.05]	−0.42, 95 % CI [−1.50, 0.67]
Waitlist arm	0.04, 95 % CI [−0.92, 0.99]	−0.92, 95 % CI [−2.37, 0.53]	−1.31, 95 % CI [−2.63, 0.01]
Diabetes management	N/A	N/A	
Treatment arm			−0.50, 95 % CI [−1.58, 0.59]
Waitlist arm			0.06, 95 % CI [−1.19, 1.30]
Diabetes QoL	N/A	N/A	
Treatment arm			−0.89, 95 % CI [−2.00, 0.22]
Waitlist arm			−0.62, 95 % CI [−1.89, 0.66]

Notes. CI: Confidence Interval. N/A: Not Applicable as T2 was not assessed.

## 4 Discussion

This was the first study to investigate the feasibility of conducting a larger trial of an online treatment with components of MSC and ACT for improving psychological distress and QoL outcomes in T2D. Considering the thresholds against which feasibility was judged, results suggested that a larger trial is not feasible due to low rates of recruitment, treatment completion, and follow-up retention. Nevertheless, the findings from this study can inform adaptations to improve the treatment and trial procedures for future research in this area.

Although the overall recruitment rate (36 %) was just below the 37 % feasibility threshold based on previous research (Glasgow et al., 2010), the sample (*N* = 33) was 82.5 % of the target sample size (*N* = 40) during the five-month recruitment window. Several issues with respect to recruitment are worth considering. For example, few participants completed the program or agreed to be interviewed. It is plausible that study advertising or participant expectations of the intervention could have influenced the poor uptake and completion in the study. Further, low recruitment may indicate that potential participants were not interested in the treatment under investigation. It is also plausible that the use of a waitlist control limited acceptability for participating, which can be addressed by including an active (e.g., CBT) or attention control in future research. It may also be that recruiting fully online, even for an online treatment, is not the optimal way to recruit participants for this type of treatment. Recruiting participants in face-to-face clinical environments may enhance the acceptability of the treatment and trial procedures. The current sample was predominantly white women. Therefore, it will be important to explore the views (e.g., through focus groups) of men with T2D and patients from diverse ethnic backgrounds about participating in research investigating the online self-compassion/ACT treatment studied here.

The treatment completion rate of 47 % was below the threshold of 57 % suggested as the average completion in previous studies of online programmes for chronic disease (Meyerowitz-Katz et al., 2020). Additionally, only 32 % of participants completed all sessions. These data suggest that the treatment was not acceptable to participants. A meta-analysis on the use of computer-based interventions for treating depression indicates that self-guided (unsupported) programmes have high drop-out (74 %) rates (Richards and Richardson, 2012). Therefore, it may be that the current online programme needs to be re-developed to integrate support elements. Such support could range from text messages, a patient support forum, or therapist input such as by telephone, as the metanalysis suggested any form of support, not only clinical, can enhance retention (Richards and Richardson, 2012). The addition of such support components may increase motivation to engage in the treatment and can be used to address potential concerns participants have about the self-compassion or ACT-based content. Additionally, a recent systematic review of primarily face-to-face group-based self-compassion therapies for people with chronic physical health conditions showed better treatment completion rates than the current study (Kılıç et al., 2021). Although that review included studies from a heterogeneous group of chronic physical health conditions, it may be that re-developing the self-compassion and ACT-based treatment studied here for face-to-face and group-based delivery would increase engagement. These suggestions for treatment re-development, either increasing support alongside the online programme or delivering in face-to-face group settings, map onto participant feedback in the current study about desiring a greater sense of social connection through the treatment.

Further consideration of the study inclusion/exclusion criteria and how these relate to engagement with treatment must also be considered. One reason for low treatment completion may be that people participating in the trial had relatively mild depression and anxiety levels and, therefore, may have been less motivated to complete treatment to manage psychological distress. However, participants did report severe levels of diabetes distress on average. Therefore, it is plausible that treatment content needed to be better tailored specifically for diabetes distress. Future large-scale trials would also benefit from using a diabetes-specific measure of distress as the primary outcome, and screening with this in the recruitment procedures, rather than more generic distress measures as diabetes distress appears to be more prevalent and relevant to participants. In addition, delivering the treatment in collaboration with a health care practitioner (e.g., a nurse) with expertise in the physiological aspects of diabetes can further enhance the treatment's tailoring by addressing patients' questions on diabetes during the treatment (e.g., Johansson et al., 2019). As self-compassion is a relatively recent area of work, participants may have struggles to see the relevance of self-compassion to their diabetes care. Working with clinicians during treatment delivery may also make integrating the treatment into routine care easier (Johansson et al., 2019), and may help participants understand the applicability of self-compassion in diabetes care. Therefore, further investigation of patients' perceptions about the use of self-compassion and ACT as treatment methods for diabetes distress, and how to facilitate engagement in these treatment approaches, is needed. Further feasibility research is also needed to enhance understanding of eligibility criteria for pre-treatment distress levels (e.g., neither too low nor too high) that may optimise engagement in this type of treatment.

The combined trial retention across arms in the current study was much lower 39 % (*n* = 13), than that observed in other trials of compassion-related treatment. For example, one previous trial reported that 77 % of participants were retained through 1-month follow up (Cheung et al., 2017). Poor retention in the treatment arm in the current study could of course be partly related to poor acceptability of treatment. Indeed, this is reflected in the lower retention (32 %) at five-weeks in the treatment arm compared to the control arm (50 %). In addition to improving treatment acceptability (as discussed above) to improve overall trial retention, it is also important to consider how other trial procedures could be improved. For example, more frequent and direct reminders (e.g., via text and telephone versus email) about the study may benefit trial retention and decrease dropouts (Richards and Richardson, 2012). Ensuring that the questionnaires are not burdensome and feel relevant to trial participants may also increase retention.

Due to significant issues with treatment completion and trial retention, the interpretation of the secondary feasibility outcomes of possible treatment effects of ACSBT-D is very challenging. Effect sizes suggest that there might be some improvements in some outcomes for those who complete the treatment. However, the number of participants providing data is very small and thus caution is warranted when interpreting these findings. To the authors' knowledge, only one other RCT of a self-compassion focused treatment has been published to date. Study findings suggested that a face-to-face group-delivered MSC programme was associated with improvements in depression, diabetes-distress and HbA1c outcomes (Friis et al., 2016). Therefore, despite the lack of feasibility in the current study, there may still be promise in pursing self-compassion treatments in people with T2D. The findings from the current study can improve treatment and trial procedures for future research in this area.

There are several limitations that need to be considered, in addition to the challenges that have already been discussed in relation to the current study and data. Firstly, the study was carried out during the COVID-19 pandemic and coincided with the UK national lockdown and later relaxations of restrictions which may have affected recruitment and retention. Secondly, a waiting list control may not have been acceptable to participants and may have limited recruitment and retention. Thirdly, given the problems with treatment completion and trial retention, the current effect size estimates must be interpreted very cautiously. Upon re-development of the treatment and trial procedures to improve acceptability, further testing is needed to explore the feasibility of a larger trial, and the current estimates should not be used to inform sample size estimates for a larger trial. Also, the trial protocol was not pre-registered. Finally, as most of the study participants were white British women, further research is needed to understand how best to engage a diversity of people with T2D in research into self-compassion and ACT for distress management.

### 4.1 Conclusion

A larger trial of the ASCBT-D programme studied here is not feasible. Further developments of treatment and trial procedures are needed to improve recruitment – including of a more diverse sample – and enhance treatment completion and trial retention rates. Findings from the current study can inform improvements of treatment and trial procedures for future research in the area of ACT and compassion-focussed treatment for psychological distress in diabetes.

## Declaration of competing interest

None.

## Data Availability

The data that support the findings of this study are not publicly available due to privacy and ethical restrictions.

## Appendix A Supplementary data

Supplementary material Image 1
