# Supplementary material for: An online acceptance, commitment, and self-compassion based treatment to decrease psychological distress in people with type 2 diabetes: A feasibility randomised-controlled trial
Source: Internet Interv. 2023 Aug 8;33:100658. doi: 10.1016/j.invent.2023.100658 (PMC10428022; doi:10.1016/j.invent.2023.100658)
Supplement: Supplementary file 1 — Supplementary material [file mmc1.docx]

Appendix A

CONSORT 2010 Checklist

| Section/Topic | Item No | Checklist item | Reported on page No |
| --- | --- | --- | --- |
| Title and abstract | | | |
|  | 1a | Identification as a randomised trial in the title | 1 |
|  | 1b | Structured summary of trial design, methods, results, and conclusions (for specific guidance see CONSORT for abstracts) | 2 |
| Introduction | | | |
| Background and objectives | 2a | Scientific background and explanation of rationale | 3 - 5 |
|  | 2b | Specific objectives or hypotheses | 4 - 5 |
| Methods | | | |
| Trial design | 3a | Description of trial design (such as parallel, factorial) including allocation ratio | 5 |
|  | 3b | Important changes to methods after trial commencement (such as eligibility criteria), with reasons | 5 |
| Participants | 4a | Eligibility criteria for participants | 5 |
|  | 4b | Settings and locations where the data were collected | 5 |
| Interventions | 5 | The interventions for each group with sufficient details to allow replication, including how and when they were actually administered | 6-8 |
| Outcomes | 6a | Completely defined pre-specified primary and secondary outcome measures, including how and when they were assessed | 8-9 |
|  | 6b | Any changes to trial outcomes after the trial commenced, with reasons | 5 |
| Sample size | 7a | How sample size was determined | 8 |
|  | 7b | When applicable, explanation of any interim analyses and stopping guidelines | N/A |
| Randomisation: |  |  |  |
| Sequence generation | 8a | Method used to generate the random allocation sequence | 5-6 |
|  | 8b | Type of randomisation; details of any restriction (such as blocking and block size) | 6 |
| Allocation concealment mechanism | 9 | Mechanism used to implement the random allocation sequence (such as sequentially numbered containers), describing any steps taken to conceal the sequence until interventions were assigned | 6 |
| Implementation | 10 | Who generated the random allocation sequence, who enrolled participants, and who assigned participants to interventions | 6 |
| Blinding | 11a | If done, who was blinded after assignment to interventions (for example, participants, care providers, those assessing outcomes) and how | N/A |
|  | 11b | If relevant, description of the similarity of interventions | N/A |
| Statistical methods | 12a | Statistical methods used to compare groups for primary and secondary outcomes | 8 -9 |
|  | 12b | Methods for additional analyses, such as subgroup analyses and adjusted analyses | N/A |
| Results | | | |
| Participant flow (a diagram is strongly recommended) | 13a | For each group, the numbers of participants who were randomly assigned, received intended treatment, and were analysed for the primary outcome | 9 - 10 |
|  | 13b | For each group, losses and exclusions after randomisation, together with reasons | 9 - 11 |
| Recruitment | 14a | Dates defining the periods of recruitment and follow-up | 5 |
|  | 14b | Why the trial ended or was stopped | 5 - 9 |
| Baseline data | 15 | A table showing baseline demographic and clinical characteristics for each group | 21 - 22 |
| Numbers analysed | 16 | For each group, number of participants (denominator) included in each analysis and whether the analysis was by original assigned groups | 9 - 11 |
| Outcomes and estimation | 17a | For each primary and secondary outcome, results for each group, and the estimated effect size and its precision (such as 95% confidence interval) | 9 – 11; 25 |
|  | 17b | For binary outcomes, presentation of both absolute and relative effect sizes is recommended | N/A |
| Ancillary analyses | 18 | Results of any other analyses performed, including subgroup analyses and adjusted analyses, distinguishing pre-specified from exploratory | N/A |
| Harms | 19 | All important harms or unintended effects in each group (for specific guidance see CONSORT for harms) | 11 |
| Discussion | | | |
| Limitations | 20 | Trial limitations, addressing sources of potential bias, imprecision, and, if relevant, multiplicity of analyses | 14 |
| Generalisability | 21 | Generalisability (external validity, applicability) of the trial findings | 11 - 15 |
| Interpretation | 22 | Interpretation consistent with results, balancing benefits and harms, and considering other relevant evidence | 11 - 15 |
| Other information | | |  |
| Registration | 23 | Registration number and name of trial registry | N/A |
| Protocol | 24 | Where the full trial protocol can be accessed, if available | N/A |
| Funding | 25 | Sources of funding and other support (such as supply of drugs), role of funders | 1 |

Appendix B

Patient partners’ recommendations for the conduct of the trial

| **Training delivery** | **Recommendations** | **Implementation** |
| --- | --- | --- |
| Length of the training | - 5 weeks or longer (*n* = 1) - 5 weeks (*n* = 4) - Less than 5 weeks (*n* = 1) | 5 weeks long programme |
| Length of time they could commit (weekly) | - More than 60 minutes (*n* = 1) - Less than 60 minutes (*n* = 1) - 40-45 minutes (*n* = 2) - 30 minutes (*n* = 1) - 20-25 mins maximum (*n* = 1) | Weekly sessions were limited to 30 minutes to balance these differing views |
| Best time for release of new material | - Before the start of the week: Sunday or Monday (*n* = 2) - Weekend (*n* = 1) - Sunday (*n* = 1) - Mid-week morning (*n* = 1) | Every Sunday around 5 pm |
| Reminders for completion of weekly sessions | - Would like to receive reminders (*n* = 6) - A reminder 48 hours before the end of the week (*n* = 1) - Reminders being sent 24 hours later if no action is taken (*n* = 1) - Two or three reminders per session (*n* = 1) | Two reminder emails were sent on Wednesdays and Fridays if no action is taken |
| **Training material** |  |  |
| Approach to information delivery | - Having written and audio versions of the material (*n* = 4) - Video or animation to deliver information (*n* = 2) - Online lectures (*n* = 1) - No preference (*n* = 1) | Information provided in written and audio formats |
| Training focus | - Tailoring to Type 2 Diabetes would be interesting but not necessary (*n* = 1) - More generic self-compassion training (*n* = 1) - Something in between diabetes specific and generic self-compassion (*n* = 1) - Specifically focusing on Type 2 Diabetes (*n* = 1) | Training focuses on targeting Type 2 Diabetes indirectly by targeting self-compassion |
| **Homework** |  |  |
| Length of time they could commit (daily) | - 15-30 minutes up to 60 minutes if it is interesting (*n* = 1) - 2 – 10 minutes (*n* = 2) - Not interested in homework practices (*n* = 1) | Homework practices are optional |
| Type of practices/tasks | - Interactive practices (*n* = 1) - Audio meditation practices (*n* = 2) | Writing practices and participants encouraged to use audio recordings for practicing on their own |
| Homework structure | - Day by day structured homework plan with some form of flexibility (e.g., providing different options) (*n* = 2) - Weekly structured plan with some form of flexibility (*n* = 1) | Weekly structured plan with different options to choose |
| **Generic** |  |  |
| Learning how to access online platform | - Video to show how you can access (n = 4) | A video provided on how to access an online platform |
| Text style, size, and colour | - Large text size (*n* = 3) - Use colours (*n* = 2) and avoid dark grey/boring screens (*n* = 1) - Use space between paragraphs (*n* = 2) - Using bold fonts and bullet points to easily track important information (*n* = 1) | These formatting suggestions were applied |
| Use of pictures and videos | - Use pictures (*n* = 3) - Use relaxing pictures such as green scenery (*n* = 1) - No preference (*n* = 1) | Requested changes were applied |

Appendix C

ACSBT-D Learning Aims

**Week 1:**

• Learning about the relationship between our mental processes and actions.

• Learning that our mental processes and actions may not always need to be in line, especially at times of distress when we need to take care of ourselves

• Discovering self-compassion and how it can help us to improve our psychological flexibility to increase our wellbeing when we go through difficult times.

**Week 2:**

• Understanding the pink fish symbol: What are values?

• Exploring our values and realising how sometimes we avoid exploring them.

• Discovering how realising our values may provoke some negative emotions, such as self-criticism or self-blame, and how self-compassion can help us to go beyond this.

**Week 3:**

• Reviewing what we have learned in the previous weeks; connecting the dots.

• Learning how we can accept difficult emotions through self-compassion.

• Learning how self-compassion can motivate us to explore new roads.

**Week 4:**

• Understanding what self-care behaviours are and why they are essential for our wellbeing

• Summarising the necessary self-care behaviours for the management of Type 2 Diabetes

• Practising these self-care behaviours through self-compassion and associating them with your values (the things you find meaningful in life).

**Week 5:**

• Overall summary of the previous weeks with an emphasis on value-based goal setting.

• Learning about gratitude, self-appreciation, savouring.

• Advice on how to continue practising self-compassion and the other concepts introduced during the training.

Appendix D

Summary of ACSBT-D content on the targeted mechanism of change through the intervention

| **Week no**  *When?* | **Content**  *Which concept will be taught?*  *From which therapy?* | **Targeted condition** |
| --- | --- | --- |
| Week 1 | Cognitive Defusion: ACT.  Self-compassion: MSC. | *Directly:*  - overall wellbeing  - distress (depression, anxiety and diabetes distress)  *Indirectly:*  - diabetes management |
| Week 2 | Values: values vs. goals, and internal and external barriers to lives based on values: ACT.  Self-criticism: MSC. | *Directly:*  - overall wellbeing  - distress  - diabetes management |
| Week 3 | Summary of previous weeks  Acceptance: ACT.  Motivation through self-compassion: MSC.  Value-based goal setting: ACT. | *Directly:*  - overall wellbeing  - distress  - diabetes management |
| Week 4 | Self-care behaviours in T2D  Self-care and value-based goal setting  S.M.A.R.T goal setting: CBT. | *Directly:*  - overall wellbeing  - distress  - diabetes management |
| Week 5 | Summary of previous weeks  Gratitude: MSC.  Self-appreciation: MSC.  Savouring: MSC. | *Directly:*  - overall wellbeing  - distress |

Note. ACT: Acceptance Commitment Therapy; MSC. Mindful Self-Compassion Course; CBT. Cognitive Behavioural Therapy.

Appendix E

Topic Guide for Semi-Structured Interviews

1. I wonder how did you find participating in this training?
   1. Was it helpful/unhelpful? If so, which aspects were the most?
   2. Which topics did you find the most helpful?
   3. Was it helpful for your diabetes management? Mental wellbeing?
   4. Were the topics covered easy to understand?
   5. What do you think about the homework material? Did you complete them? If so, how much and how long?
   6. Would you recommend this to any other person?
2. What do you think about the delivery of this training?
   1. How did you find online participation? (Individual vs group settings)
   2. Length of the training?
   3. Communication with the researcher?
3. What do you think about the possible contribution of this training in your life? (optional)

Appendix F

Selected outcome measures

**Depression.** The Patient Health Questionnaire-8 (PHQ-8; Kroenke et al., 2009), consists of eight questions rated on a four-point scale (0 = Not at all; 3 = Nearly every day) used to assess depressive mood. Scoring ten or more indicates the probability of depression (Kroenke et ak., 2009). The scale was chosen due to being frequently used in the UK primary care and diabetes population (van Dijk et al., 2018).

**Anxiety.** The Generalised Anxiety Disorder-7 questionnaire (GAD-7; Spitzer, Kroenke, Williams, & Löwe, 2006) is advised by Diabetes UK as a well-validated measure of generalised anxiety (Hendrieckx, Halliday, Beeney, & Speight, 2019). The scale includes seven questions rated on a four-point scale (0 = Not at all; 3 = Nearly every day). Scoring five, ten or fifteen indicates the probability of mild, moderate, and severe anxiety levels (Spitzer et al., 2006).

**Diabetes-distress.** The Problem areas in Diabetes questionnaire (PAID; Welch, Jacobson, & Polonsky, 1997) is one of the most commonly used questionnaires to measure diabetes specific distress (Dennick, Sturt, & Speight, 2017). The PAID includes questions around diabetes management which distinguishes it from more general anxiety and depression questionnaires. The PAID includes 20 questions rated on a five-point scale (0 = Not a problem; 4 = Serious problem). Scoring 40 or more means severe diabetes distress (Welch et al., 1997).

**Generic wellbeing.** The Well-Being Questionnaire (12 items) (W-BQ12) (W-BQ12; Bradley, 1994) measures general wellbeing. The scale includes 12 questions rated on a four-point Likert scale (0 = Not at all; 3 = All the time). The higher scores indicate higher general wellbeing. The scale has been previously used in diabetes studies (e.g., Pouwer, Snoek, van der Ploeg, Adèr, & Heine, 2001).

**Diabetes quality of life.**The Audit of Diabetes Dependent Quality of Life 19 questionnaire (ADDQoL19; Bradley et al., 1999) is an individualised measure of the perceived impact of diabetes on quality of life (QoL). The questionnaire includes 19 QoL domains, such as leisure, work, journeys, holidays, physical, family life and others based on its impact and importance. While impact questions are rated on a five-point scale (-3 to 1), importance questions are rated on a four-point scale (3 to 0). A total score ranging between -9 to 3 produced where negative scores indicate negative impact or vice versa (Bradley et al., 1999).

**Diabetes Self-Management.** The Diabetes Self-Management Questionnaire (DSMQ Schmitt et al., 2013) assesses self-management relating to glycaemic control in common treatment regimens for people with diabetes. The scale includes 16 items rated on a four-point scale (0 = Does not apply to me; 3 = Applies to me very much). The scale has demonstrated reliability and for self-care behaviours linked to glycaemic control (Schmitt et al., 2013).

**Self-compassion.** The Self-Compassion Scale (SCS; Neff, 2003; Neff et al., 2019) measures compassion towards the self-using 13 positively and 13 negatively worded questions, each rated on a five-point scale (1 = Almost Never; 5 = Almost Always). The SCS has previously been used in the diabetes population (e.g., Friis, Johnson, Cutfield, & Consedine, 2016). Negative items are reverse-scored and higher total scores there indicate greater self-compassion.

**Psychological inflexibility.** The Acceptance and Action Questionnaire-2 (AAQ-2; Bond et al., 2011) was used to measure psychological inflexibility, reflecting the ineffective struggle to control or avoid emotions at the expense of one’s goals and values. The scale includes seven questions rated on a seven-point scale (1 = Never True; 7 = Always True). The scale has also been used previously in diabetes studies (e.g., Nicholas, Yeap, Cross, & Burkhardt, 2021). Higher scores indicate greater psychological inflexibility.

References

Bond, F. W., Hayes, S. C., Baer, R. A., Carpenter, K. M., Guenole, N., Orcutt, H. K., . . . Zettle, R. D. (2011). Preliminary Psychometric Properties of the Acceptance and Action Questionnaire–II: A Revised Measure of Psychological Inflexibility and Experiential Avoidance. *Behavior Therapy, 42*(4), 676-688. doi:<https://doi.org/10.1016/j.beth.2011.03.007>

Bradley. (1994). *The well-being questionnaire*: Chur, Switzerland, Harwood Academic Publishers.

Bradley, Todd, C., Gorton, T., Symonds, E., Martin, A., & Plowright, R. (1999). The development of an individualized questionnaire measure of perceived impact of diabetes on quality of life: the ADDQoL. *Quality of Life Research, 8*(1), 79-91. doi:10.1023/A:1026485130100

Dennick, K., Sturt, J., & Speight, J. (2017). What is diabetes distress and how can we measure it? A narrative review and conceptual model. *Journal of Diabetes and its Complications, 31*(5), 898-911.

Friis, A., M., Johnson, Cutfield, & Consedine. (2016). Kindness matters: a randomized controlled trial of a mindful self-compassion intervention improves depression, distress, and HbA1c among patients with diabetes. *Diabetes care, 39*(11), 1963-1971.

Hendrieckx, C., Halliday, J., Beeney, L., & Speight, J. (2019). Diabetes and Emotional Health: A Practical Guide for Healthcare Professionals Supporting Adults with Type 1 or Type 2 Diabetes. *London: Diabetes UK: National Diabetes Services Scheme (NDSS)*.

Kroenke, K., Strine, T. W., Spitzer, R. L., Williams, J. B., Berry, J. T., & Mokdad, A. H. (2009). The PHQ-8 as a measure of current depression in the general population. *Journal of Affective Disorders, 114*(1-3), 163-173.

Neff. (2003). The development and validation of a scale to measure self-compassion. *Self and Identity, 2*(3), 223-250. doi:10.1080/15298860309027

Neff, Tóth-Király, I., Yarnell, L. M., Arimitsu, K., Castilho, P., Ghorbani, N., . . . Mantzios, M. (2019). Examining the factor structure of the Self-Compassion Scale in 20 diverse samples: Support for use of a total score and six subscale scores. *Psychol Assess, 31*(1), 27-45. doi:10.1037/pas0000629

Nicholas, J. A., Yeap, B. B., Cross, D., & Burkhardt, M. S. (2021). Psychological flexibility is associated with less diabetes distress and lower glycated haemoglobin in adults with type 1 diabetes. *Internal Medicine Journal*.

Pouwer, F., Snoek, F. J., van der Ploeg, H. M., Adèr, H. J., & Heine, R. J. (2001). Monitoring of Psychological Well-Being in Outpatients With Diabetes. *Diabetes care, 24*(11), 1929. doi:10.2337/diacare.24.11.1929

Schmitt, A., Gahr, A., Hermanns, N., Kulzer, B., Huber, J., & Haak, T. (2013). The Diabetes Self-Management Questionnaire (DSMQ): development and evaluation of an instrument to assess diabetes self-care activities associated with glycaemic control. *Health and quality of life outcomes, 11*(1), 138. doi:10.1186/1477-7525-11-138

Spitzer, R. L., Kroenke, K., Williams, J. B., & Löwe, B. (2006). A brief measure for assessing generalized anxiety disorder: the GAD-7. *Archives of internal medicine, 166*(10), 1092-1097.

van Dijk, S. E. M., Adriaanse, M. C., van der Zwaan, L., Bosmans, J. E., van Marwijk, H. W. J., van Tulder, M. W., & Terwee, C. B. (2018). Measurement properties of depression questionnaires in patients with diabetes: a systematic review. *Quality of Life Research, 27*(6), 1415-1430. doi:10.1007/s11136-018-1782-y

Welch, G. W., Jacobson, A. M., & Polonsky, W. H. (1997). The Problem Areas in Diabetes Scale: an evaluation of its clinical utility. *Diabetes care, 20*(5), 760-766.

Appendix G

Content analysis of open-ended feedback on ACSBT-D

| *Most helpful aspects:* | *Least helpful aspects:* | *What to improve:* |
| --- | --- | --- |
| Self-compassionate perspective for diabetes management  (*n* = 2) | Lacking face-to-face delivery  (*n* = 1) | Providing a space for interacting with others  (*n* = 2) |
| Well-structured and easy to understand  (*n* = 2) | Lack of sustainability and accountability of a group-based delivery  (*n* = 1) | Increasing the length of sessions for more advanced practitioners of self-compassion  (*n* = 1) |
| Interesting topics (e.g., values, linking self-compassion and diabetes)  (*n* = 2) | Computer-based participation felt distant and lack of belonging  (*n* = 1) | Applying mixed mode of deliveries (e.g., group settings letting also personalised practice)  (*n* = 1) |
| Being present and accountable for what you do (*n* = 2) | Some training materials are too easy for people who already practice mindfulness  (*n* = 1) |  |
| Adequate time to complete each session  (*n* = 2) |  |  |
| Valuable exercises/practices  (*n* = 1) |  |  |
| Time/space for reflection, especially on self-compassion  (*n* = 1) |  |  |
| Mixed presentation of the learning material  (*n* = 1) |  |  |
| Practising regularly  (*n* = 1) |  |  |
| Encouraged to do what I want  (*n* = 1) |  |  |
